# Clinical and radiographic outcomes of 19 proximal thoracic pedicle subtraction osteotomies for adult spinal deformity: a case series

**DOI:** 10.1007/s43390-025-01076-1

**Published:** 2025-03-20

**Authors:** Justin Bird, Maxwell M. Scott, Christopher Lucasti, Benjamin C. Graham, David Kowalski, Emily K. Vallee, Danielle E. Chipman, Dil V. Patel, Christopher L. Hamill

**Affiliations:** 1 Orthopaedics and Sports Medicine, UBMD, University at Buffalo, Buffalo, NY USA; 2 Jacobs School of Medicine and Biomedical Sciences, University at Buffalo, 955 Main St, Buffalo, NY 14203 USA

**Keywords:** Pedicle subtraction osteotomy, Thoracic, Spinal deformity, Fixed sagittal deformity, Sagittal alignment, Complications, Radiographic outcomes, Case series

## Abstract

**Purpose:**

To present a detailed analysis of postoperative clinical and radiographic outcomes of patients who underwent proximal thoracic pedicle subtraction osteotomy (PSO) for adult spinal deformity.

**Methods:**

A retrospective chart review was performed on 19 patients who underwent proximal thoracic (T2–T4) PSO between January 2018 and December 2021. Baseline patient characteristics, complications and radiographic outcomes were collected. Radiographic outcomes including thoracic kyphosis correction, overall segment correction, and global sagittal balance correction were measured using preoperative and postoperative radiographs.

**Results:**

19 patients with an average age of 66.9 ± 8.3 years underwent thoracic PSO, with 94.7% (*n* = 18) being females, in the setting of revision surgery. The mean thoracic kyphosis correction was 20.4 ± 8.5°. Overall segmental correction had a mean of 16.2 ± 3.9°. Global sagittal balance correction was an average of 13.9 ± 23.2 mm (mm). The median hospital stay was 4.0 (IQR: 3.0) days with a median of 1.0 (IQR: 2.0) days in the intensive care unit. 36.8% (*n* = 7) of patients had a major complication within 30 days: proximal junction kyphosis (PJK) (2), neurologic deficits (2), pneumonia (1), cardiopulmonary (1), death (1). 47.4% (*n* = 9) of patients had a major complication within 2 years: PJK (5), neurologic deficits (2), wound dehiscence/infection (1), pneumonia (1), cardiopulmonary (1), death (2). Average follow up was 636 (range: 43–1320).

**Conclusion:**

While thoracic PSO can achieve successful radiographic and clinical outcomes, it is also associated with a high risk of potential major complications and mortality, such as instrumentation or junctional failure and neurologic deficits.

**Graphical abstract:**

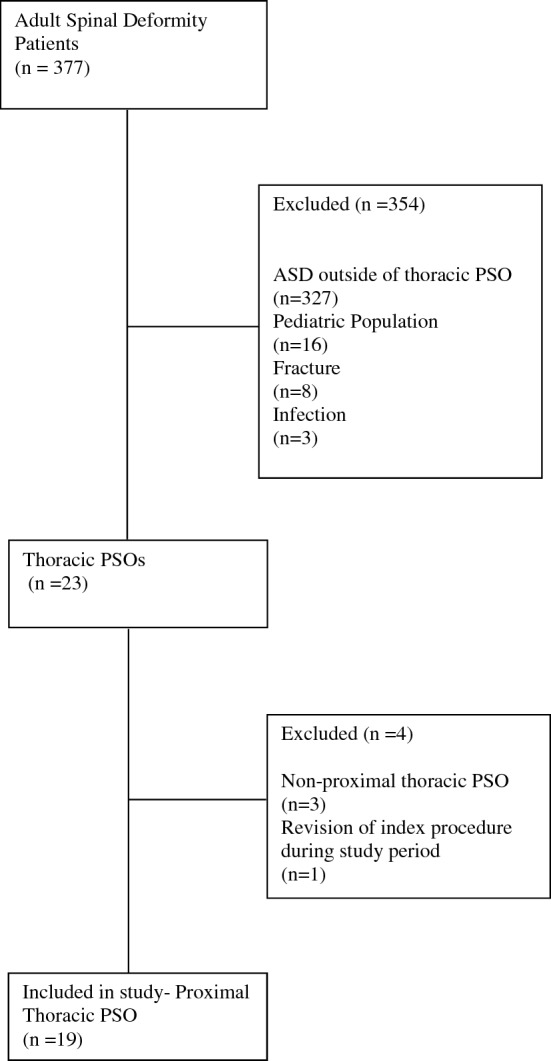

## Introduction

Pedicle subtraction osteotomy (PSO) is a surgical intervention that has gained popularity for its correction of fixed sagittal deformity, particularly in the distal thoracic or lumbar spine. Additionally, in certain instances, a proximal thoracic PSO (levels T2–T4) can be performed. Prior to its development, surgical management of severe spinal deformity traditionally involved a 360° anterior and posterior approach [1, 2]. The main benefit of PSO compared to these traditional methods includes its ability to maximize correction in the coronal and sagittal planes while avoiding some of the deleterious effects of the anterior approach [3–5]. Additional benefits of PSO include its potential to introduce up to 40° of lordotic correction in the setting of fixed kyphotic deformities [6, 7].

While the PSO can offer significant corrections in patients with severe spinal deformities, it also has notable disadvantages. PSOs are technically challenging and often involve prolonged operative times and increased blood loss relative to other posterior-based procedures. These technical challenges and extended operative times put patients at risk for various complications such as major blood loss, neurological deficits, deep wound infections, pulmonary embolus, myocardial infarction, and others [8–12].

PSO is typically utilized in the distal thoracic or lumbar regions of the spine, especially in cases of iatrogenic flatback deformity and global kyphoscoliosis. However, the fixed kyphotic deformity may be present in the proximal thoracic spine, and in such cases, a thoracic PSO can be utilized to correct the deformity [13]. Proximal thoracic PSOs present greater technical challenges compared to distal thoracic or lumbar PSOs, primarily due to the heightened risk of spinal cord injury. [14].

While numerous studies have published the outcomes and complications associated with lumbar PSOs, there is a paucity of literature describing complications and radiographic improvements following proximal thoracic PSO surgery. The purpose of this case series is to provide radiographic outcomes and complications in patients undergoing proximal thoracic PSO at our institution.

## Materials and methods

### Data collection

After Institutional Review Board approval (IRB 00006727), data was collected by retrospective chart review of patients who underwent proximal thoracic PSO surgery at a single institution by a single fellowship-trained orthopedic spine surgeon with over twenty-five years of experience. All surgeries were performed under fluoroscopic guidance, without the use of navigation or robotics. A total of 23 thoracic PSO surgeries were performed between January 1, 2018, and December 31, 2021, with 19 meeting the inclusion criteria: > 18 years old, non-revision of index procedure during the study period, who underwent primary or revision proximal thoracic PSO. Exclusion criteria included infectious causes of the index procedure, non-proximal thoracic PSO, and tumor-related causes.

Baseline demographic variables included age, gender, body mass index (BMI), and smoking status. Comorbidities included cardiovascular disease (CVD), coronary artery disease (CAD), hypertension (HTN), hypercholesterolemia (HCL), and diabetes mellitus (DM). Additionally, the Charlson Comorbidity Index (CCI) and American Society of Anesthesiologist Class (ASA) were calculated for each patient. Surgical factors collected included the history of spinal fusion, operating room time, PSO level, number of fusion levels, use of Smith-Peterson osteotomy (SPO), estimated blood loss (EBL), and intraoperative complications.

All pre- and postoperative radiographic measures were conducted utilizing Picture Archiving and Communication System (PACS) software with an included measuring tool. All measurements were taken by a senior orthopedic surgery resident. Preoperative measurements were taken at the most recent x-ray prior to surgery. Postoperative X-rays were collected at the last follow-up. Segmental kyphosis was measured utilizing the Cobb angle of the superior endplate of the vertebrae above the PSO level and the inferior endplate of the vertebrae below the PSO level. Thoracic kyphosis was measured at the superior endplate of T1 to the inferior endplate of T12. Sagittal vertical goal axis was measured using the distance between the C7 plumb line and the posterior superior sacral endplate.

The outcomes recorded were 30-day and 2-year complication rates and readmission rates, thoracic kyphosis correction, overall segment correction, and global sagittal balance correction. When considering complications, we looked for those previously described by Glassman et al. [15]. When considering junctional failure, any patient with radiologic evidence of proximal junctional kyphosis was considered a major complication. Additional outcome measures included hospital length of stay, intensive care unit (ICU) length of stay, 30-day and 2-year infection rates, and discharge disposition.

### PSO technique

Patients were positioned prone on a Jackson table with Gardner-Wells tongs used for traction. Standard 3-foot anterior–posterior and lateral X-rays were obtained, and localizing images were used to map the incision based on the patient’s deformity and existing instrumentation. A standard posterior subperiosteal dissection was used to expose the instrumentation. Native lateral mass screws were utilized in the subaxial cervical spine. At the C2 level, pedicle screws were used, and all screws in the thoracic spine were pedicle screws. The PSO level was chosen at the surgeon’s preference and was performed at the apex of the deformity. All patients underwent PSO with no vertebral column resections, Smith-Peterson osteotomies, or cages. Smith-Peterson osteotomies were considered, but at the surgeon’s discretion, none were performed. SRS-Schwab Classification was noted to be 3 for all 19 patients included in this study.

The PSO was first performed by removing the lamina of the level where the PSO was to be performed, followed by laminectomies at levels above and below. Facetectomies were performed at the levels above and below to expose the pedicle at the level of PSO. Two cm of rib and rib head were resected bilaterally to allow for lateral access to the vertebral body at the PSO level. The remainder of the bilateral pedicles were removed with a burr while protecting the thecal sac. Next, a retractor was placed along the lateral aspect of the vertebral body on one side to protect the thoracic cavity structures and an osteotome was used to create a wedged resection of the posterior vertebral body. A temporary rod was placed ipsilaterally, and the wedged resection was performed on the contralateral side. Corrective maneuver was sequential compression across osteotomy sites with direct force applied to the spine and placement of the two rods. The osteotomy site was closed using rod compressors and the remaining rods were cut, contoured, and placed according to the patent’s level and extent of deformity. In some cases, the osteotomy site was stabilized using dual rod fixation; however, in later years of this series, the surgeon transitioned to using quad rod fixation as the preferred approach. All rods were 5.5 cobalt chrome. The upper instrumented vertebrae varied based on the patient specific deformity (Table 5).

The hemostatic protocol consisted of a standard bolus dose of 10 to 20 mg/kg administered prior to skin incision, followed by a maintenance infusion of 1 to 10 mg/kg per hour throughout the surgical duration, along with the use of a cell saver in all cases.

### Data analysis

A Shapiro–Wilk test for normality was performed on all continuous variables. Therefore, normally distributed continuous variables are reported as mean and standard deviation (SD), and non-normally distributed variables are reported as median and interquartile range (IQR). Continuous variables were recorded as averages with ranges. Categorical variables were reported as percentages. Pre- and postoperative radiographs were analyzed to measure segmental thoracic and global kyphosis and global sagittal balance. The average segmental and global kyphosis correction as well as the average change in global sagittal balance was calculated. All radiographic measurements were performed using PACS software using radiographic measuring tools, while data analysis was performed using Microsoft Excel (Version 16.79.2) and SPSS Statistical Analysis Software, Version 29 (IBM, Armonk, NY, USA).

## Results

### Demographics

19 patients with a minimum 2-year follow-up were included in the study. The average age of our cohort is 66.9 ± 8.3 years, and most patients were female (94.7%, *n* = 18). 52.6% (*n* = 10) of patients were non-obese, and 84.2% (*n* = 16) were non-smokers. Most patients did not have a history of DM, CVD, HTN, or dyslipidemia (Table 1). Patients had CCI scores between 0 and 5 and ASA classes between 2 and 4 (Table 1).

**Table 1:** Demographic characteristics of entire cohort (*N* = 19)

	Mean ± SD
Age (years)	66.9 ± 8.3
	*N* (%)
Gender	
Female	18 (94.7)
Male	1 (5.3)
Body Mass Index (BMI) (kg/m2)	
Non-Obese (BMI 25.0–29.9 kg/m^2^)	10 (52.6)
Obese (BMI ≥ 30.0 kg/m^2^)	9 (47.4)
Smoking Status	
Non-smoker	16 (84.2)
Smoker	3 (15.8)
Diabetes Status	
Non-diabetic	17 (89.5)
Diabetic	2 (10.5)
Cardiovascular Disease (CVD) Status	
No CVD	14 (73.7)
CVD	5 (26.3)
Hypertension Status	
No Hypertension	10 (52.6)
Hypertension	9 (47.4)
Cholesterol Status	
No hypercholesterolemia	11 (57.9)
Hypercholesterolemia	8 (42.1)
CCI Score	
0	1 (5.3)
1	2 (10.5)
2	5 (26.3)
3	6 (31.6)
4	3 (15.8)
5	2 (10.5)
ASA Class	
1	0 (0)
2	1 (5.3)
3	17 (89.5)
4	1 (5.3)

*CCI* Charlson Comorbidity Index, *ASA* American Society of Anesthesiologist

### Presentation and surgical details

All patients had a history of a previous spinal fusion surgery. 68.4% (*n* = 13) of patients presented with junctional kyphosis, 26.3% (*n* = 5) had posttraumatic kyphosis, and 5.3% (*n* = 1) of patients presented with degenerative kyphoscoliosis. The median number of levels fused was 8.0 (IQR: 4.0) levels. All PSOs were in the proximal thoracic spine from T2 to T4 (Table 5). The median estimated blood loss was 825.0 (IQR: 552.0) milliliters (mL). Patients had a mean thoracic kyphosis correction of 20.4 ± 8.5° (Table 4). Overall segmental correction had a mean of 16.2 ± 3.9°. Global sagittal balance correction was an average of 13.9 ± 23.2 mm (mm) (Table 2).

**Table 2:** Perioperative Characteristics, Length of Stay, and Readmissions of Entire Cohort (*N* = 19)

	*N* (%)
Prior Spinal Fusion	
Yes	19 (100.0)
Deformity Etiology	
Junctional Kyphosis	13 (68.4)
Posttraumatic Kyphosis	5 (26.3)
Degenerative Kyphoscoliosis	1 (5.3)
Thoracic PSO Level	
Proximal Thoracic (T2-4)	19 (100.0)
Discharge	
Home	5 (26.3)
SAR	13 (68.4)
Death	1 (5.3)
Readmission	
30 days	2 (10.5)
90 days	2 (10.5)
	Median (IQR)
Number of Levels Fused	8.0 (4.0)
OR Time (minutes)	305.0 (81.0)
EBL (mL)	825.0 (552.0)
Hospital Length of Stay (days)	4.0 (3.0)
Intensive Care Unit Length of Stay (days)	1.0 (2.0)

*PSO* pedicle subtraction osteotomy, *OR* operating room, *EBL* estimated blood loss, *SAR* subacute rehabilitation

### Hospital course, complications, readmissions and discharge disposition

Following thoracic PSO surgery, the median hospital length of stay was 4.0 (IQR:3) days with a median of 1.0 (IQR: 2) days in the ICU. 36.8% (*n* = 7) of patients had a major complication within 30 days: proximal junctional kyphosis (PJK) (2), Neurologic deficits (2), Pneumonia (1), Death (1), Cardiopulmonary complication (1). Within two years of the index procedure, 47.4% of patients (*n* = 9) had a major complication: PJK (5), Neurologic deficits (2), Death (2), Wound dehiscence/infection (1), Pneumonia (1), Cardiopulmonary (1) (Tables 3, 4).

**Table 3:** Complications of Entire Cohort (*N* = 19)

	*N* (%)
Intraoperative Complications	
Neuromonitoring Change	1 (5.3)
Pedicle Breach	1 (5.3)
Perioperative Complications within 30 days	
Instrumentation/Junctional Failure	2 (10.5)
Pneumonia	1 (5.3)
Neurological Complications	2 (10.5)
Other Cardiopulmonary	1 (5.3)
Death	1 (5.3)
Postoperative Complications within 2 years*	
Instrumentation/Junctional Failure	5 (26.3)
Neurologic Complications	2 (5.3)
Death	2 (10.5)
Pneumonia	1 (5.3)
Wound Dehiscence/Infection	1 (5.3)
Other Cardiopulmonary	1 (5.3)

*Table includes patients with multiple complications

**Table 4:** Proximal thoracic PSO: sagittal correction

	Mean ± SD
Thoracic Kyphosis Correction (°)	20.4 ± 8.5
Overall Segmental Correction (°)	16.2 ± 3.9
Global Sagittal Balance Correction (mm)	13.9 ± 23.2

For patients with PJK, three of them needed revision surgery due to failure of the instrumentation. Additionally, one of the patients with PJK also developed a wound dehiscence that required surgical intervention. The first patient who expired experienced a standard postoperative course before suffering pulseless electrical activity on postoperative day five leading to death, despite being neurologically intact (Table 3). A second patient died outside of the 30-day postoperative window. This patient faced a major neurological complication due to instrumentation failure requiring revision surgery. Following surgery, she experienced a challenging postoperative period that ultimately resulted in death on postoperative day 32 due to cardiac arrest and respiratory failure. The patient with a cardiopulmonary complication developed bilateral pleural effusions postoperatively on day 3. The patient with pneumonia developed respiratory compromise on post-operative day 10, most likely secondary to an aspiration event. Finally, the second patient with motor deficits was doing well postoperatively but had a fall during her rehabilitation that resulted in traumatic spondylolisthesis. She developed bilateral leg weakness and an inability to ambulate requiring revision surgery. After revision surgery, she regained sensation in her legs and some lower extremity strength but remained unable to ambulate.

Most patients were discharged to a subacute rehabilitation center (68.4%, *n* = 13), with the rest being discharged home (26.3% *n* = 5). 10.5% (*n* = 2) of patients were readmitted within 30 days of discharge, and 21.1% (*n* = 4) were readmitted within 90 days of discharge.

## Case example

**Fig. 1:**
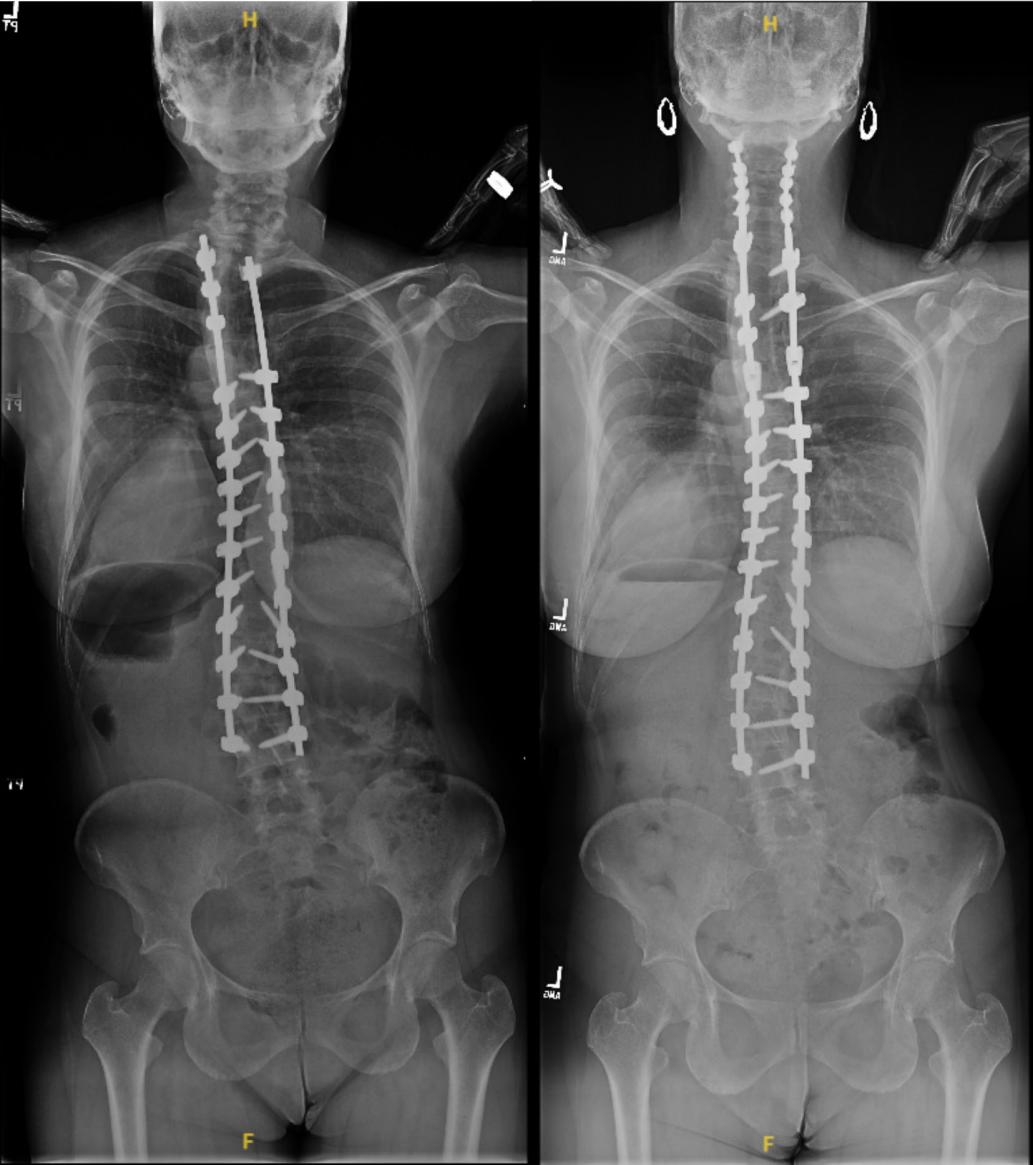
Standing Coronal Scoliosis Films of Patient 13 Pre-Operative (Left) and at Four Year Follow-Up (Right)

**Fig. 2:**
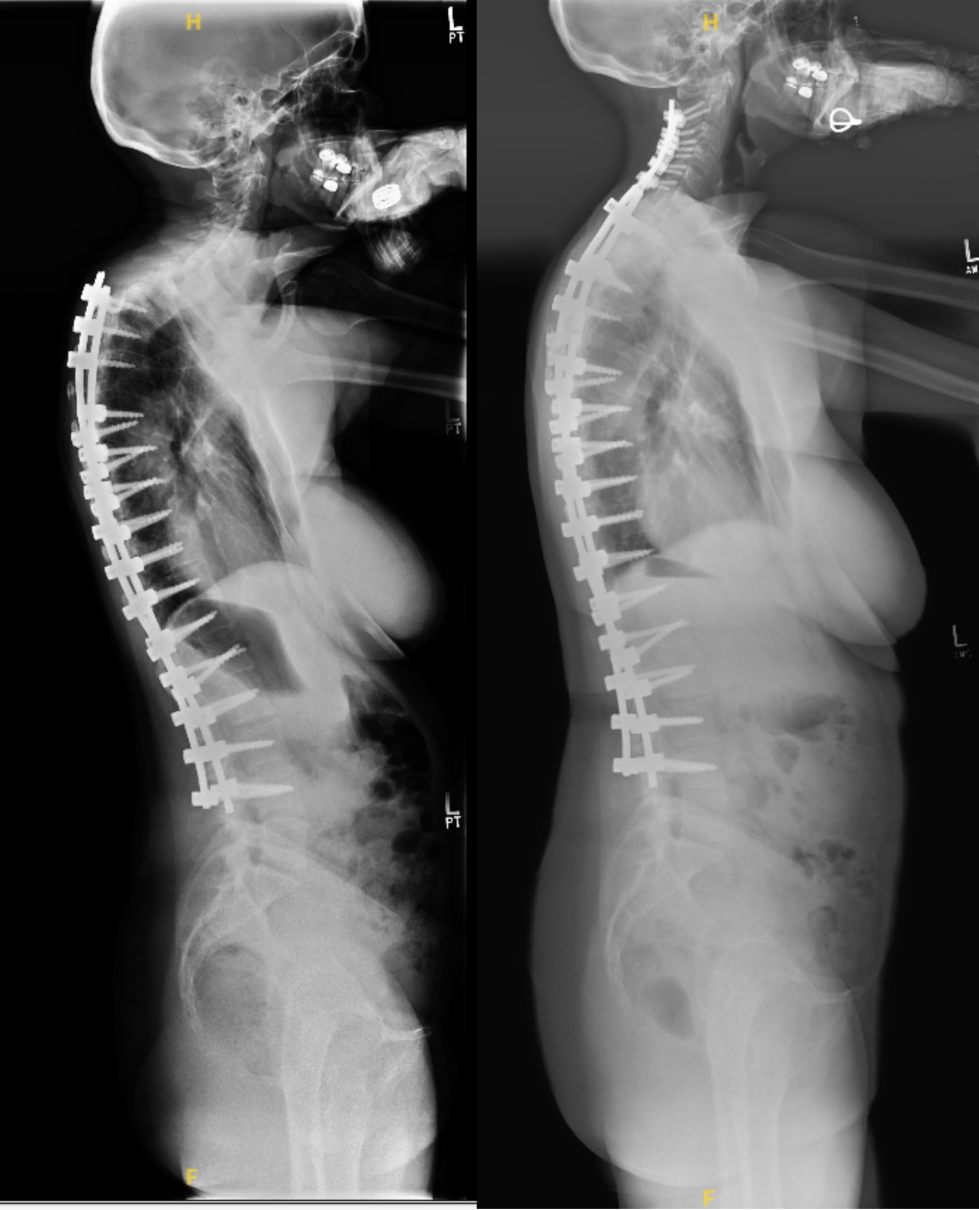
Standing Sagittal Scoliosis Films of Patient 13 Pre-Operative (Left) and at Four Year Follow-Up (Right)

A 48-year-old female presented to the office with anteroposterior and lateral radiographs demonstrating prior posterior decompression instrumented fusion T2–L4 at an outside institution. The patient subsequently developed chin-on-chest deformity secondary to proximal junctional kyphosis (Fig. 1). The patient elected to undergo revision posterior decompression instrumented fusion C2-T7 with a pedicle subtraction osteotomy at T2. Her preoperative global kyphosis was 78° and postoperatively 59° for a global correction of 19° (Figs. 1 and 2). Preoperative segmental kyphosis was 39° and postoperatively 26° for a segmental correction of 13°. Postoperative anteroposterior and lateral radiographs at final follow-up 4 years status post revision demonstrate maintenance of coronal and sagittal alignment (Figs. 1 and 2). A summary of patient characteristics can be found in Table 5 (Patient 13).

**Table 5:** Summary of Patient Characteristics (*N* = 22)

Patient	Age/ Gender	Etiology	PSO Level	Surgery (UIV BOLDED UNDERLINED)	Complications	Thoracic Kyphosis Correction	Segmental Correction	Global Sagittal Balance Correction	LOS/ Discharge
1	79; female	Posttraumatic Kyphosis	T3	Partial ROH, **C6**-T8 PF, T3 PSO	Instrumentation/ junctional failure (fracture at UIV C6)	17°	20°	15 mm	4(1); SAR
2	72; female	Junctional Kyphosis	T3	Partial ROH, **T1**-T8 PF, T3 PSO	–	17°	11°	50 mm	4(1); SAR
3	67; female	Posttraumatic Kyphosis	T3	**T1**-8 PF, T3 PSO	Instrumentation/ junctional failure (Fracture subluxation at C7-T1)	9°	13°	10 mm	4(1); Home
4	75; female	Junctional. Kyphosis	T4	ROH, **C2**-Pelvis PF, T4 PSO C2-C7 Laminectomy	Pneumonia	38°	17°	60 mm	30(11); SAR
5	78; male	Posttraumatic Kyphosis	T3	**T1**-11 PF, T3 PSO	Instrumentation/ junctional failure (chin on chest deformity); wound dehiscence	37°	26°	5 mm	6(4); SAR
6	72; female	Junctional Kyphosis	T3	**T1**-7 PF, T3 PSO	Neurological complications; Death	21°	13°	30 mm	33(32); death
7	65; female	Junctional Kyphosis	T3	**C2**-T10 PF, T3 PSO	Death	–	–	–	5(3); Death
8	58; female	Posttraumatic Kyphosis	T3	Partial ROH, **T1**-T9 PF, T4 PSO	Instrumentation/ junctional failure (Fracture subluxation C7-T1)	19°	12°	15 mm	3(0); SAR
9	72; female	Junctional Kyphosis	T4	Partial ROH, **C2**-T8 fusion, T4 PSO	-	16°	16°	30 mm	7(2); SAR
10	74; female	Degenerative Kyphoscoliosis	T3	**T1**-8 PF, T3 PSO	Instrumentation/ junctional failure (Chin on chest deformity/ rod fracture in Lumbar Spine)	23°	20°	25 mm	7(3); SAR
11	61; female	Junctional Kyphosis	T3	ROH, **C3**-T7 PF, T3 PSO	–	28°	19°	10 mm	4(1); Home
12	57; female	Junctional Kyphosis	T4	**T1**-9 PF, T4 PSO	–	30°	15°	25 mm	3(1); Home
13	49; female	Junctional Kyphosis	T2	ROH, **C2**-T7 PF T2 PSO,	–	19°	13°	5 mm	3(2); Home
14	62; female	Posttraumatic Kyphosis	T4	Partial ROH, **T2**-T12 PF, T4 PSO,	Motor deficit	8°	16°	20 mm	6(3); SAR
15	68; female	Junctional Kyphosis	T3	Partial ROH, **T1**-T8 PF, T3 PSO	–	9°	12.5°	30 mm	4(1); Home
16	68; female	Junctional Kyphosis	T3	Partial ROH, **T1**-T7 PF, T3 PSO,	Cardiopulmonary	19°	20°	25 mm	7(3); SAR
17	73; female	Posttraumatic Kyphosis	T3	Partial ROH, **T1**-T6 fusion, T3 PSO,	–	18°	17°	25 mm	4(1); SAR
18	67; female	Junctional Kyphosis	T3	Partial ROH, **T1**-T8 fusion, T3 PSO,	–	23°	13°	5 mm	3(1); SAR
19	54; female	Posttraumatic Kyphosis	T3	Partial ROH, **T1**-T9 fusion, T3 PSO	–	17°	18°	15 mm	(7)1; SAR

*PF* posterior fusion, *PSO* Pedicle subtraction osteotomy, *ROH* Removal of hardware, *SPO* Smith-Peterson osteotomy, *mm* millimeter, *SAR* Subacute Rehabilitation, *UIV* Upper Instrumented Vertebrae

## Discussion

Thoracic PSO is a complex and powerful surgical technique that can be utilized in cases of severe thoracic spinal deformity. Currently, they are performed on a limited basis in the United States due to high complication rates and limited correction compared to lumbar PSOs. Even more infrequent is proximal thoracic PSO. Given the infrequency of these surgeries, this case series sought to provide information on 19 proximal thoracic PSO patients within a five-year period. We provide information on demographics, deformity etiology, surgical details, surgical correction, hospital course, complications, and readmissions for 19 patients undergoing thoracic PSO surgery.

The average age of patients in our case series was 66.9 ± 8.3 years and most of the patients were female (94.7%, *n* = 18). Obeid et al. reported on 10 cases of proximal thoracic PSO and found a younger average age of 40.7 years [14]. Additionally, they reported more male patients than female (50% male, *n* = 5/10). O’Shaughnessy et al. also found a younger average age of 56 years with a lower percentage of females (60%, *n* = 9/15) [13]. Theologies et al. and Cacho-Rodrigues et al. also reported a younger age for thoracic osteotomies with less female patients (59 years, 60.6% (*n* = 20/33) male and 44 years, 64.7% (*n* = 11/17) male) [16, 17]. Zeng, et al. also reported that most upper thoracic three-column osteotomies occurred in younger patients [18]. In our study, all PSO occurred between T2 and T4.

Our study found the median hospital length of stay was 4.0 (IQR:3) days with a median of 1.0 (IQR: 2) days in the ICU. Theologis et al. had a similar ICU stay at 2.9 days, but a longer total hospitalization at 9.7 days [17]. Obeid et al. found a longer average hospital length of stay at 13.8 days [14]. Bakaloudis et al. examined thoracic PSOs in 12 adolescents and found an average hospital length of stay of 10.4 days and an ICU stay of 1.5 days [19]. Overall, our results reinforce the postoperative high acuity care that is required with multiple days in the hospital for these technically demanding and higher morbidity thoracic osteotomies.

At our institution, 36.8% of patients undergoing a proximal thoracic PSO experienced a complication within 30 days of the procedure. Faundez et al. reported a similar complication rate of 46.4% (*n* = 13/28) [20]. In studies focused on proximal thoracic PSO, there is a similar complication rate of 50% (*n* = 5/10) [14]. In our case series, the most common complication at two years postoperative was instrumentation/junctional failure (26.3% *n* = 5). O’Shaughnessy et al. reported that 20% (*n* = 3/15) of PSOs had early structural complications [13]. Another 4 (27%) patients had structural complications outside of the first 30 days [13]. This is similar to the 26.3% of patients in our cohort who had instrumentation or junctional failure within two years. Obeid et al. reported that 10% of patients had instrumentation failure within 30 days, similar to our rate of 5.3% (*n* = 1) within 30 days of the operation [14]. Other non-structural intraoperative complications have been documented in the literature, such as pneumothorax, dural tears, dysphagia, need for tracheostomy, and postoperative nerve root palsy [17]. However, in this study, we found no such incidences, which reiterates the importance of surgeon experience to mitigate intraoperative and postoperative complications. We did have on patient experience pneumonia secondary to an aspiration event. This is a possible complication with the elderly and stiffer necks. At our institution, we monitor patients perioperatively for swallowing difficulties, encourage sitting up to eat, and involve speech and language if there is any concern. One 12-year study found that surgeon experience and higher case numbers, not number of three-column osteotomies performed, was associated with lower perioperative complication rate and faster total surgical time [21].

Patients in our study had an overall segmental correction of 16.2 ± 3.9°. O’Shaughnessy et al. found an overall segmental correction of 16.3° when examining thoracic PSOs from the entire thoracic spine [13]. Obeid et al. found a segmental correction of 26.0° when focusing on proximal thoracic PSO [14]. Faundez et al. found a segmental correction of 17.5° when examining PSOs from T1–T5, and 18.2° when examining T6–T9 [20]. Additionally, we found a thoracic kyphosis correction of 20.4 ± 8.5°. When compared to similarly sized studies, our results show a similar correction (12.6° to 29.5°) [13, 14, 17, 19]. Lastly, our global sagittal correction was 13.9 ± 23.2 mm. Past studies have found a range of correction from 6.73 mm to 23.0 mm [13, 17].

Despite the value of this case series, it is not without limitations. First, as a retrospective chart review, it is inherently limited by selection bias. Second, there is limited clinical outcome data in our study as no disability scores were utilized. Additionally, given the infrequent use of thoracic PSOs, only 19 patients are reported in this case series. Also, this study only examines complications within two years of surgery with not all patients having followed up beyond the two-year mark. Lastly, all surgeries were conducted by a single spine fellowship-trained surgeon with 25 years of experience, so the generalizability of these results is unclear.

## Conclusion

Thoracic PSOs are a valuable surgical option for correcting severe thoracic kyphotic deformities. It allows for significant correction of global and thoracic kyphosis as well as global sagittal balance. This study contributes to the minimal existing body of research on proximal thoracic PSOs and demonstrates the expected postoperative outcomes when this powerful surgical technique is appropriately selected for use. Proper patient selection, meticulous surgical planning, and precise execution are essential for achieving successful outcomes. However, it is important to understand that these surgeries are associated with high morbidity and mortality. Advancements in surgical techniques and technology continue to enhance the safety and effectiveness of this procedure, offering improved options for patients with challenging spinal deformities. Nevertheless, potential complications underscore the importance of a careful risk–benefit assessment and the need for ongoing research in this field given the high morbidity and mortality of thoracic PSOs.

## Data Availability

The data supporting the findings of this study are available from the corresponding author upon reasonable request.
